# New method for selection of hydrogen peroxide adapted bifidobacteria cells using continuous culture and immobilized cell technology

**DOI:** 10.1186/1475-2859-9-60

**Published:** 2010-07-27

**Authors:** Valeria Mozzetti, Franck Grattepanche, Déborah Moine, Bernard Berger, Enea Rezzonico, Leo Meile, Fabrizio Arigoni, Christophe Lacroix

**Affiliations:** 1 Laboratory of Food Biotechnology, Institute of Food Science and Nutrition, Schmelzbergstrasse 7, ETH-Zurich, 8092 Zürich, Switzerland; 2 Nestlé Research Center, Vers-chez-les-Blanc, 1000 Lausanne 26, Switzerland

## Abstract

**Background:**

Oxidative stress can severely compromise viability of bifidobacteria. Exposure of *Bifidobacterium *cells to oxygen causes accumulation of reactive oxygen species, mainly hydrogen peroxide, leading to cell death. In this study, we tested the suitability of continuous culture under increasing selective pressure combined with immobilized cell technology for the selection of hydrogen peroxide adapted *Bifidobacterium *cells. Cells of *B. longum *NCC2705 were immobilized in gellan-xanthan gum gel beads and used to continuously ferment MRS medium containing increasing concentration of H_2_O_2 _from 0 to 130 ppm.

**Results:**

At the beginning of the culture, high cell density of 10^13 ^CFU per litre of reactor was tested. The continuous culture gradually adapted to increasing H_2_O_2 _concentrations. However, after increasing the H_2_O_2 _concentration to 130 ppm the OD of the culture decreased to 0. Full wash out was prevented by the immobilization of the cells in gel matrix. Hence after stopping the stress, it was possible to re-grow the cells that survived the highest lethal dose of H_2_O_2 _and to select two adapted colonies (HPR1 and HPR2) after plating of the culture effluent. In contrast to HPR1, HPR2 showed stable characteristics over at least 70 generations and exhibited also higher tolerance to O_2 _than non adapted wild type cells. Preliminary characterization of HPR2 was carried out by global genome expression profile analysis. Two genes coding for a protein with unknown function and possessing trans-membrane domains and an ABC-type transporter protein were overexpressed in HPR2 cells compared to wild type cells.

**Conclusions:**

Our study showed that continuous culture with cell immobilization is a valid approach for selecting cells adapted to hydrogen peroxide. Elucidation of H_2_O_2 _adaptation mechanisms in HPR2 could be helpful to develop oxygen resistant bifidobacteria.

## Background

According to FAO/WHO (2002) [1], probiotics are defined as "live microorganisms which when administered in adequate amounts confer a health benefit on the host". One of the crucial points in the production and distribution of probiotic foods is hence to deliver enough live probiotic cells to the consumers. The minimum daily intake of probiotics to obtain a beneficial effect is still under debate. However, a concentration of 10^6 ^live cells of probiotic bacteria per gram of product at the time of consumption is generally accepted and selected to provide bacterial concentrations that are attainable and cost-effective for probiotic food products [2]. During production and storage of food, microorganisms experience a wide range of stresses, including oxidative stress, which can severely compromise cell viability of sensitive strains as well as their incorporation into food products.

Bacterial strains belonging to *Bifidobacterium *and *Lactobacillus *genera are the most widely used microorganisms in probiotic food products and supplements [3]. Bifidobacteria are obligate anaerobes. However, some species can tolerate oxygen, such as *B. psychroaerophilum*, *B. indicum *and *B. asteroides*, the latter two possessing catalase [4,5]. Exposure to oxygen induces accumulation of reactive oxygen species, mainly hydrogen peroxide, which cause oxidative damage to vital cellular components, resulting in cell death of sensitive cells [6,7].

Although oxygen tolerant bifidobacteria can be isolated from the environment [8], these isolates do not necessarily exhibit relevant probiotic characteristics. Mutagenic agents have also been applied to obtain oxygen resistant bifidobacteria [9]. However, random mutations affecting the probiotic characteristics of the strain may occur. Another method to isolate stress resistant bacterial strains consists in culturing cells in presence of a selective agent. Plating and cultivation in batch cultures with varying concentration of a selective agent are fairly simple procedures, but the number of generations over which selection can occur is limited. Therefore repeated cycles of subculturing may be required. In contrast to batch, continuous culture can be performed over an unlimited number of generations under strictly controlled conditions. Moreover, it can ensure continuous presence of unstable selective agents, such as hydrogen peroxide that can be broken up into nascent oxygen and water in contact with organic matter of rich media like MRS broth [10]. However, the use of continuous culture combined with selective pressure is limited because the resistance level of cells can greatly vary with fermentation time. In addition, an over-dosage of the selective agent leads generally to a wash-out of cells from the reactor. This major drawback of continuous cultures can be prevented using immobilized cell technology, which allows retaining cell in reactor even if dilution rate exceed growth rate of the culture [2,11].

The aim of this study was to test the application of continuous culture with immobilized cells for selecting hydrogen peroxide adapted populations of *B. longum *NCC2705.

## Methods

### Bacterial strain

*B. longum *NCC2705 was obtained from the Nestlé Culture Collection (Lausanne, Switzerland) and cultivated in MRS [12] medium (Biolife, Milano, Italy). Two successive pre-cultures, inoculated at 1% from a frozen stock at -80°C in MRS with 10% glycerol (Sigma-Aldrich, Buchs, Switzerland), were performed for 16 h under anaerobic (AnaeroGen, Oxoid, Basingstoke, United Kingdom) conditions at 37°C before use.

### Cell immobilization

Cell immobilization was based on a two-phase dispersion process as previously described [13]. A mixed gel of 2.5% (w/v) gelrite gellan gum and 0.25% (w/v) xanthan gum (both Sigma-Aldrich) was inoculated at 2% (v/v) with a pre-culture of *B. longum *NCC2705, containing ca. 10^9 ^CFU ml^-1^. Beads with diameters in the range of 1.0-2.0 mm were selected by wet sieving and used for fermentation. The entire process was completed within 1 h.

### Continuous culture

A volume of 70 ml of inoculated gel beads was transferred into a 1-l stirred tank reactor (Multifors, Infors-HT, Bottmingen, Switzerland) containing 630 ml MRS. The reactor was stirred at 100 rpm by an inclined blade impeller. Nitrogen was aseptically injected into the headspace of the reactor to maintain anaerobic conditions. Temperature was set at 37°C and pH was controlled and maintained at 6.0 by adding 5 M NaOH. Culture was started in batch mode for the first 24 h, followed by 24 h in continuous mode with feeding of MRS at 2.6 ml min^-1^, using a peristaltic pump (Infors-HT), to allow colonization of gel beads. During this colonization step, immobilized population increased from 10^7 ^to 10^11 ^CFU g^-1 ^of gel beads. Afterwards, feeding of H_2_O_2 _solutions was started, using a calibrated peristaltic pump (Infors-HT), and the culture was carried out in continuous mode for 23 days. Inflow rate of MRS, initially set to 2.6 ml min^-1 ^was decreased to 0.9 ml min^-1 ^at day 9. H_2_O_2 _was continuously added to the reactor at a flow rate 10 fold smaller than that of the MRS and using concentrated solutions ranging from 50 to 1,300 ppm depending on the applied H_2_O_2 _level. H_2_O_2 _solutions were prepared using 30% H_2_O_2 _(VWR, Dietikon, Switzerland) in sterile water. H_2_O_2 _solution was kept on ice, protected from light and replaced daily, to avoid H_2_O_2 _breakdown during the experiment. Effluent samples were collected from the reactor at different time intervals for optical density (600 nm) measurements using sterile MRS medium as reference, and cell enumeration using plate counts. Aliquots of 2 ml effluent samples were centrifuged (6,000 g for 2 min) and pellets suspended in equal volume of fresh MRS containing 10% glycerol and stored at -80°C for further analyses. Gel bead samples of 1 g were placed into 1 ml of MRS with 10% glycerol and stored at -80°C until analysis.

### Viable cell enumeration in culture effluent and gel beads

Samples from fermented effluent were serially diluted in phosphate buffered saline (pH 7.7) supplemented with 0.05% L-cysteine hydrochloride monohydrate (Sigma-Aldrich) (C-PBS). Appropriate dilutions were plated in duplicate on MRS agar (DIFCO, Becton Dickinson AG, Allschwil, Switzerland) and incubated anaerobically at 37°C for 48 h. The immobilized cell population was also monitored by plate counts after adding ca. 0.5 g gel beads to 20 ml 1% EDTA (Sigma-Aldrich) and treatment in a stomacher (Seward, Norfolk, UK) for 3 min for bead dissolution before dilution in C-PBS and plating.

### H_2_O_2 _resistance level of cells from culture effluent

Resistance to H_2_O_2 _over fermentation time of cells from culture effluent was tested as follows. Cells from frozen samples were twice subcultured in MRS inoculated at 1% (v/v) and incubated anaerobically at 37°C for 24 h. Aliquot of 1 ml containing ca. 1.11 ± 0.67 × 10^9 ^CFU ml^-1 ^was centrifuged at 6,000 g for 2 min and the pellet was suspended in 10 ml 400 ppm H_2_O_2 _solution. After 1.5 h incubation at room temperature, the cell suspension was diluted in C-PBS and plated on MRS agar and incubated anaerobically at 37°C for 48 h. Results were expressed as survival rate in percent before and after treatment. The test was performed in duplicate.

### Isolation of cells adapted to H_2_O_2_

Frozen bead samples (1 g) collected at day 18 of continuous culture, were dissolved in 40 ml 1% EDTA, treated in a stomacher for 3 min and centrifuged at 6,000 g for 5 min. Cell pellets were suspended in 10 ml 40 ppm H_2_O_2 _and incubated for 60 min at room temperature to recover fractions of populations adapted to H_2_O_2 _stress. 1 ml of cell suspension was subsequently plated on MRS agar. Colonies visible within 48 h were twice subcultured anaerobically for 16 h in MRS broth at 37°C and frozen at -80°C in MRS with 10% glycerol until further analyses.

### Characterization of H_2_O_2 _adapted isolates

#### H_2_O_2 _resistance level of isolates

Frozen samples of wild type and H_2_O_2 _adapted isolate cultures containing approximately 1.03 ± 0.33 × 10^9 ^CFU ml^-1 ^were thawed at room temperature. 1 ml of sample was centrifuged at 6,000 g for 2 min. The pellet was suspended in 10 ml 200 ppm H_2_O_2 _solution. After 2 h incubation at room temperature cell suspensions were diluted in C-PBS and plated on MRS agar and incubated anaerobically at 37°C for 48 h. Results were expressed as survival rate in percent before and after treatment. The test was performed in triplicate.

#### Stability of H_2_O_2 _adapted phenotype

Stability of H_2_O_2 _adapted phenotype of isolates was tested by subculturing cells without selective pressure in MRS at 37°C under anaerobic conditions for 24 h. After each subculture, containing ca. 1.76 ± 1.07 × 10^9 ^CFU ml^-1^, resistance level to H_2_O_2 _of cells was tested using 400 ppm H_2_O_2 _solution as described above. The test was performed in duplicate.

#### Growth in presence of oxygen in liquid shaking cultures

Ability of cells to grow in presence of oxygen was tested according to Meile *et al. *[8] with some modifications. The headspace of 500 ml serum flasks containing 400 ml MRS, after creating vacuum conditions was flushed with N_2 _or CO_2 _until normal atmosphere pressure was achieved; then 7.5 or 12.5% (v/v) sterile oxygen were added. Afterwards, the medium was inoculated at 2% with an overnight culture of wild type or H_2_O_2 _resistant cells and the flasks were incubated at 37°C in a shaker at 160 rpm (Kühner AG, Basel, Switzerland) for 24 h. Samples were taken every 2 h during the first 12 h of the culture to measure optical density at 600 nm. Two repetitions were carried out.

#### Growth in reactor with and without H_2_O_2_

Cells were grown in an 800-ml working volume reactor (1-l reactor, Infors-HT) containing MRS inoculated at 5% with an overnight culture of wild type or H_2_O_2 _resistant cells. Temperature was controlled at 37°C and agitation set at 200 rpm. Anaerobic conditions were maintained by continuously sparging either CO_2 _or N_2 _in the medium, starting overnight prior inoculation. Cells were cultivated with and without addition of 42 ppm H_2_O_2 _in mid-exponential growth phase corresponding to an OD of 0.6. This H_2_O_2 _concentration of 42 ppm causes a cessation in growth for at least 40 min of exponentially wild type cells of *Bifidobacterium longum *NCC2705 without a decrease in viable cell counts during this growth arrest [14]. Growth was monitored by measuring OD at 600 nm. Samples for global transcriptional profiling were taken in mid-exponential growth phase after 3-3.5 h of culture at an OD of 0.7-0.8 in reactors sparged with CO_2 _and without H_2_O_2_. Aliquots of 2 ml were centrifuged (4,000 g for 1 min), supernatants discarded and cell pellets snap frozen in liquid nitrogen and stored at -80°C until RNA-extraction. Fermentations were performed in triplicate.

### Microarray analysis

#### Microarray design and RNA extraction

DNA based arrays, produced by Agilent Technologies http://www.agilent.com, were obtained by *in situ *synthesis of 60 mer oligonucleotides on glass slides [15]. For each gene, 3 to 6 different probes were randomly distributed on the array. Total RNA was extracted with the macaloïd method and purified as previously described [16].

#### Array hybridization

For each hybridization, cDNA was synthesized starting from 4 μg of total RNA and subsequently labeled using the Array 900MPX Genisphere kit (Genisphere Inc., Hatfield, PE, USA), following the protocol provided by the supplier. Luciferase and kanamycin control mRNA (Promega, Zürich, Switzerland) at 1 and 10 ng, respectively, were mixed with total RNA before labeling to allow balancing of the two channels during scanning. After the hybridization procedure, array slides were scanned at 10 μm using a Scanarray 4000 (Packard Biochip Technologies, Billerica, MA, USA). Laser power and photomultiplier tube gain were set in order to prevent saturation of any spot, except the probes corresponding to rRNA.

#### Array analysis

Data extracted with Imagene 5.6 (Biodiscovery, El Segundo, CA, USA) were treated with homemade scripts in Python language http://www.python.org and a local installation of the ArrayPipe web server [17]. Probes showing a signal smaller than twice the standard deviation of the local background were considered without signal. Probes showing no signal or saturated signals in both channels were discarded from the analysis. Assuming an intensity-dependent variation in dye signal, (limma) loess global normalization was applied on signal ratios. To calculate average gene expression values, data from 3 biological replicates were combined as follows. Within each hybridization data set, gene fold changes were calculated from the median of the corresponding probes values. The expression value of a gene was retained if a signal was detected in at least 50% of its probes. Genes were considered to be differentially expressed if their log_2_-transformed signal ratios were higher than 1.5 or smaller than -1.5. Statistical analysis of the 3 biological array replicates of the hybridization between wild type and H_2_O_2 _resistant cells were performed using the statistical software R version 2.6.1 [18]. Bayes statistics for differential expression [19] was used to rank genes in order of evidence for differential expression. The data have been deposited in NCBI's Gene Expression Omnibus and are accessible through GEO Series accession number GSE16039. TMHMM-prediction server version 2.0 was used to identify transmembrane domains of predicted proteins [20].

## Results

### Continuous culture monitoring

**Figure 1:**
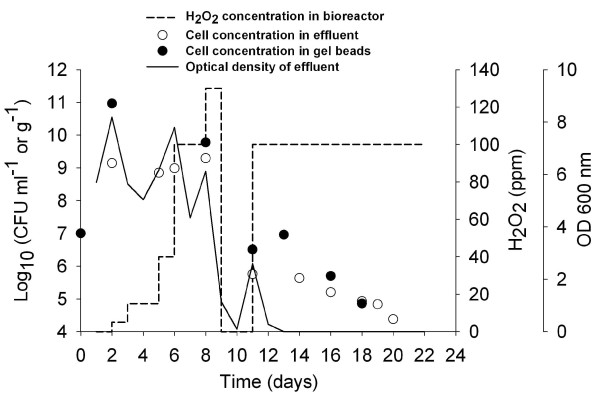
**Continuous culture monitoring**. The figure shows the optical density of fermented effluent, H**_2_**O**_2 _**concentration in bioreactor, viable cells counts in gel beads and in the effluent during continuous culture with immobilized cells of ***B. longum ***NCC2705 and H**_2_**O**_2 _**selective pressure.

The concentration of H_2_O_2 _in the continuous culture with immobilized cells of *B. longum *NCC2705 was increased stepwise to 130 ppm in order to select for cells resistant to oxidative stress (Figure 1). At day 2, optical density of the fermented effluent reached 8.4, corresponding to 1.26 × 10^9 ^CFU ml^-1^. Subsequently, OD decreased and increased after each increase in H_2_O_2 _concentration. Viable cells in effluent samples before changing the level of H_2_O_2 _concentration ranged from 6.31 × 10^8 ^to 2.00 × 10^9 ^CFU ml^-1 ^until day 8 (Figure 1). Following H_2_O_2 _concentration increase to 130 ppm at day 9, the OD decreased to 0. To enrich potentially hydrogen peroxide resistant survivors after this harsh treatment, the addition of H_2_O_2 _was stopped for 2 days and the flow rate of the medium was decreased to 0.9 ml min^-1^, resulting in a new phase of growth with an increase of OD to 3.2 at day 11 in effluent samples, corresponding to 6.31 × 10^5 ^CFU ml^-1^. Concentration of H_2_O_2 _was then set again at 100 ppm and OD decreased again to 0 with viable cell counts in the effluent gradually decreasing from 3.98 × 10^5 ^at day 14 to 2.51 × 10^4 ^CFU ml^-1 ^at day 20 (Figure 1). Cell counts in gel beads were generally 1 to 2 log higher than in effluent samples, except at the end of culture where both population reached similar levels (Figure 1).

**Figure 2:**
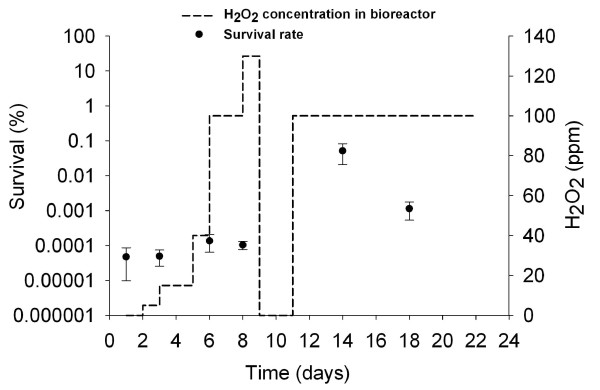
**Resistance of cells collected from fermented effluent to H_2_O_2_**. Resistance of cells from fermented effluent to H**_2_**O**_2 _**as a function of fermentation time was assessed after incubation in 400 ppm H**_2_**O**_2 _**solution for 1.5 h and expressed in % survival. Applied concentrations of H**_2_**O**_2 _**in the bioreactor are also indicated. Data are means of two replicates ± standard deviation.

H_2_O_2 _resistance of free cells in the culture effluent remained stable during the first 8 days with a survival rate of 0.00010 ± 0.00004% (Figure 2). At day 14 and 18 the survival rate was 760 and 16 folds higher than for day 1, respectively (Figure 2).

### Selection of resistant cells and stability of H_2_O_2 _resistance phenotype

**Figure 3:**
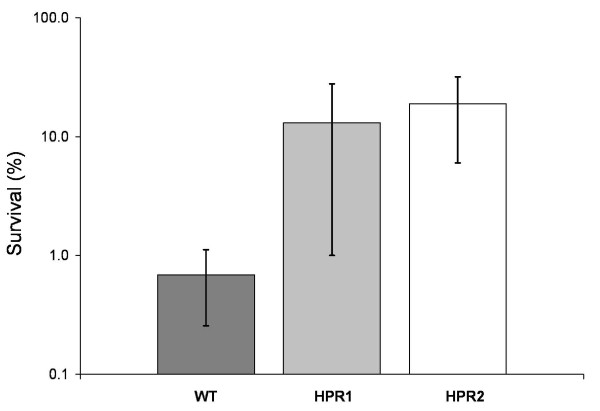
**Resistance level of isolates HPR1 and HPR2 and of wild type (WT) *B. longum *NCC2705 to H_2_O_2_**. Resistance was assessed after incubation of cells in 200 ppm H_2_O_2 _solution for 2 h and expressed in % survival. Data are means of three replicates ± standard deviation.

**Figure 4:**
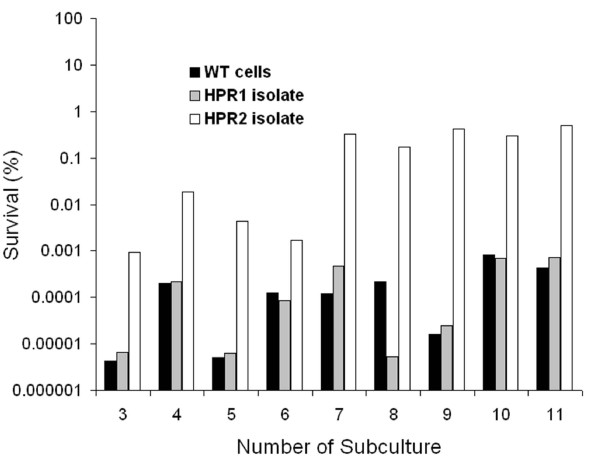
**Stability of H_2_O_2 _adapted phenotype of isolates HPR1 and HPR2**. The figure shows the survival (in %) to 2 h, 400 ppm H_2_O_2 _solution of wild type, and of H_2_O_2 _resistant phenotype HPR1 and HPR2 cells after successive subcultures without selective pressure.

Because a large fraction of cell biomass of the system was in the bead matrix (ca. 10^11 ^CFU g^-1 ^beads after colonization) at the beginning of the fermentation, a higher number of adapted cells was expected in the gel beads, which were physically retained in the reactor, than in effluent at the end of culture. Cells from gel beads collected at day 18 (6.31 × 10^4 ^CFU g^-1^) were subjected to an isolation step, and tested for their resistance to H_2_O_2_. Two colonies, namely HPR1 and HPR2, were detected after this isolation step. Tolerance to H_2_O_2 _of cells from these two isolates was 20-and 30-folds higher, respectively, than that of wild type cells (Figure 3). A stability test was performed with these two isolates using a hydrogen peroxide concentration of 400 ppm and incubation time of 2 hours. After three successive subcultures, HPR1 cells exhibited similar resistance level to wild type cells, whereas HPR2 maintained its phenotype over at least 11 subsequent cultures (Figure 4). The survival tests showed large variations depending on the testing day; however a repetition of this test showed similar trends (data not shown).

### Growth in presence of oxygen in liquid shaking cultures

**Figure 5:**
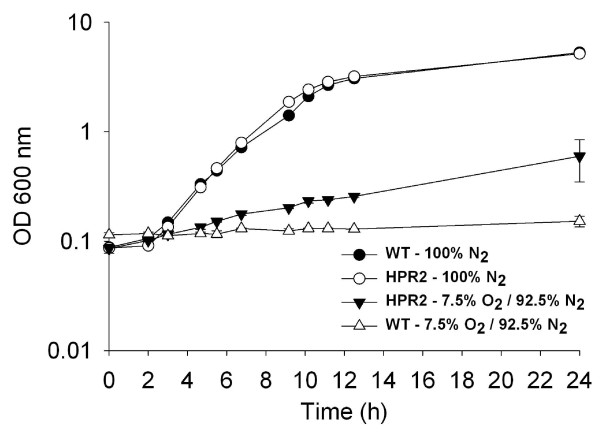
**Growth of HPR2 and wild type *B. longum *NCC2705 in presence of 100% N_2 _and of 7.5% O_2 _-92.5% N_2_**. Cells were grown in liquid shaking cultures. Growth was monitored using optical density at 600 nm. Data are means of two replicates ± standard deviation.

**Figure 6:**
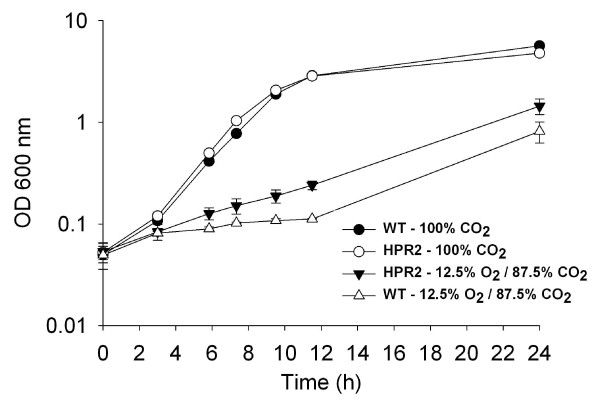
**Growth of HPR2 and wild type *B. longum *NCC2705 in presence of 100% CO_2 _and of 12.5% O_2 _-87.5% CO_2_**. Cells were grown in liquid shaking cultures. Growth was monitored using optical density at 600 nm. Data are means of two replicates ± standard deviation.

HPR2 and wild type cells grew at similar rate of 0.47 ± 0.03 h^-1 ^and reached the same optical density of 5.22 ± 0.36 after 24 h culture in presence of 100% nitrogen or carbon dioxide in the headspace (Figures 5 and 6). When the atmosphere of the headspace was composed of 7.5 and 92.5% of oxygen and nitrogen, respectively, HPR2 cells grew at a lower rate of 0.09 ± 0.00 h^-1 ^and reached an OD of 0.60 ± 0.02 after 24 h culture while growth of wild type cells was negligible with a growth rate of 0.01 ± 0.00 h^-1 ^and a final OD of 0.15 ± 0.02 (Figure 5). Using a headspace of 12.5 and 87.5% of oxygen and carbon dioxide, respectively, the growth rate during the first 11.5 h was 0.12 ± 0.01 and 0.04 ± 0.01 h^-1 ^for HPR2 and wild type cells, respectively (Figure 6). From 11.5 to 24 h of culture, both strains grew at similar rate of 0.15 ± 0.01 h^-1 ^and reached a final OD of 1.44 ± 0.25 and 0.81 ± 0.19 for HPR2 and wild type cells, respectively (Figure 6).

### Growth in presence of H_2_O_2 _in reactors

**Figure 7:**
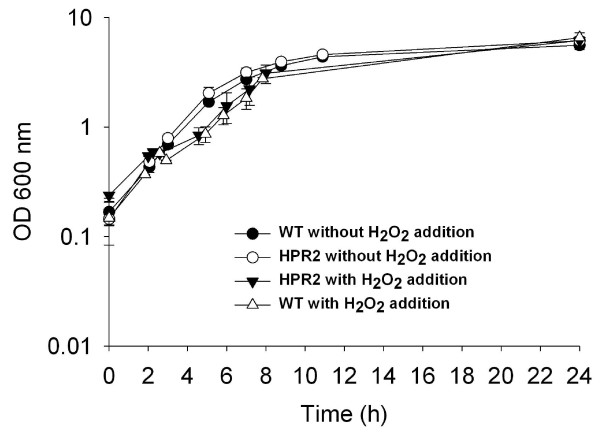
**Growth of HPR2 and wild type *B. longum *NCC2705 in bioreactors under CO_2 _atmosphere, with and without addition of H_2_O_2_**. Cells were cultivated under CO_2 _atmosphere and with or without addition of 42 ppm H_2_O_2 _in mid-exponential growth phase corresponding to an OD of 0.6. Growth was monitored using optical density at 600 nm. Data are means of three replicates ± standard deviation.

**Figure 8:**
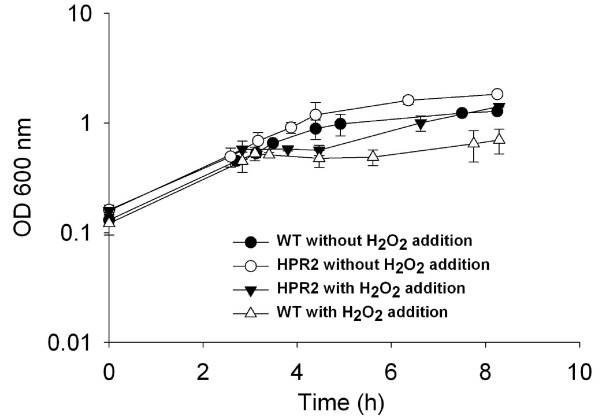
**Growth of HPR2 and wild type *B. longum *NCC2705 in bioreactors under N_2 _atmosphere, with and without addition of H_2_O_2_**. Cells were cultivated under N_2 _atmosphere and with or without addition of 42 ppm H_2_O_2 _in mid-exponential growth phase corresponding to an OD of 0.6. Growth was monitored using optical density at 600 nm. Data are means of three replicates ± standard deviation.

Both wild type and HPR2 cells recovered rapidly and in a similar manner after addition of H_2_O_2 _in medium sparged with carbon dioxide, and reached the same OD of 6.06 ± 0.60 than non treated cells after 24 h of culture (Figure 7). In presence of nitrogen, HPR2 strain started to recover earlier (1.63 vs. 2.51 h after addition of hydrogen peroxide) and grew at a higher rate (0.24 ± 0.02 vs. 0.13 ± 0.03 h^-1^) than wild type cells after H_2_O_2 _addition, reaching an optical density of 1.41 ± 0.02 and 0.70 ± 0.18, respectively, after 8 h culture (Figure 8). Growth of HPR2 and wild type cells, treated or not with H_2_O_2_, were negatively affected by sparging nitrogen compared to carbon dioxide in the medium (Figures 7 and 8).

### Genome-wide transcription analysis

Transcriptome analysis of exponentially growing cells without selective pressure showed that in HPR2 compared to wild type cells, the genes *BL1404 *and *BL0931 *were overexpressed. *BL1404 *was over-expressed with an average log_2 _ratio of 3.1 (2.3-3.8, 95% confidence interval) and 1.5 (1.6-1.7, 95% CI) for *BL0931*. The start codon of *BL0931 *gene is 45 nucleotide downstream of *BL0932 *stop codon, a gene encoding an ABC-transporter ATP-binding protein. Both genes are predicted to be on the same operon, a prediction which is supported by the similar average gene expression profile displayed by the *BL0932 *gene (1.4; 0.9-1.9, 95% CI). All remaining genes had log_2 _transformed ratios lower than 1.5 and non significant p-values.

## Discussion

In this study, we tested the suitability of continuous culture under increasing selective pressure combined with immobilized cell technology for the selection of hydrogen peroxide adapted bifidobacteria cells. Continuous system allows cultivation of cells over an unlimited number of generations under strictly controlled conditions. In addition continuously cultured cells may undergo a number of consecutive mutational events, each contributing to improve adaptation of cells to their environment [21,22]. Evolutionary engineering approaches using chemostats or repeated batch cultures, combined with constant or gradually increasing selective pressure have been successfully exploited to increase resistance of industrially relevant microorganisms, mainly yeast cells, to environmental stresses [23-25].

A very high cell concentration was reached in beads (10^11 ^CFU g^-1 ^beads) leading to a high total population, including immobilized and free cells, of 10^13 ^CFU per litre of reactor similar to that reported by Doleyres *et al. *[26] with immobilized *B. longum *cells in gellan gum beads during continuous culture operated at a dilution rate of 0.5 h^-1^. As a comparison, batch cultures with free cells of *B. longum *NCC2705 generally reach 10^9 ^CFU ml^-1^, corresponding to 10^12 ^CFU per litre of reactor at the end of the culture (data not shown). This high cell density could favor the occurrence of mutations since the rate of appearance of mutational events is proportional to the amount of biomass in the culture [27].

The continuous culture gradually adapted to increasing H_2_O_2 _concentrations, as shown by the culture oscillating OD. However, the *B. longum *population tested in the effluent reached an upper limit of its adaptive capabilities at day 9 with H_2_O_2 _concentration of 130 ppm. Immobilization of the cells in gel matrix has prevented full wash-out, which would have very likely occurred with a free cell system. Hence after stopping the stress, it was possible to re-grow the cells that survived this high H_2_O_2 _concentration. After this enrichment step and under 100 ppm H_2_O_2_, resistance level of continuously produced cells was higher than those tested at the beginning of the fermentation but decreased to an intermediate level at day 18. Heterogeneity in the immobilized and free populations which can exhibit different resistance level and/or adaptive mechanisms to the selective pressure, as discussed below for cells isolated from gel beads, could explain this change in resistance to H_2_O_2 _with fermentation time and using the same conditions. Çakar *et al. *[25] reported a heterogeneous resistance levels to cobalt for single cells within an evolved population of *Saccharomyces cerevisiae *subjected to continuous increasing levels of cobalt stress.

HPR1 and HPR2, isolated from gel beads at day 18, showed a 20-and 30-fold higher resistance to H_2_O_2 _than wild type cells, respectively. Two different mechanisms can be proposed to explain the adaptive response of these two isolates in regard to the stability of their phenotype. HPR1 isolate lost rapidly its hydrogen peroxide adapted phenotype which could be the result of a transient adaptation caused by the stressing conditions encountered in gel beads and not necessarily by H_2_O_2 _selective pressure. Indeed, it was already reported that immobilized *B. longum *cells, continuously cultivated without specific selective pressure adapt to H_2_O_2 _stress [28]. This phenotype was reversible after subculturing, and could be related to a non specific stress adaptation caused by diffusional limitations of both substrates and inhibitory products in the gel beads [29]. Resistance phenotype of HPR2 isolate to H_2_O_2 _was much more stable (at least over 70 generations) than that of HPR1 indicating a probable stable mutation. To our knowledge, this is the first description of a mutant strain of bifidobacteria resistant to H_2_O_2_. HPR2 cells were then further characterized.

HPR2 could tolerate higher O_2 _level than wild type cells of *B. longum *NCC2705 and the moderately oxygen-tolerant *B. thermophilum *RBL67 as reported by von Ah *et al. *[29]. The ability of HPR2 isolate to grow in presence of oxygen can be associated with its H_2_O_2 _adapted phenotype. Indeed, accumulation of H_2_O_2 _during culture of sensitive bifidobacteria in presence of oxygen is generally considered as the primary reason for growth inhibition [7,30,31]. Additionally, HPR2 and wild type cells seem to tolerate more O_2 _in presence of CO_2 _than with N_2 _in the headspace of the culture. This result is different from that of Kawasaki *et al. *[30] who reported that colony development of different *Bifidobacterium *species under an atmosphere composed of 5 and 95% O_2 _and N_2_, respectively, was not improved by addition of CO_2_. This difference can be explained by the methodology and strains used in our studies. The better growth of HPR2 and wild type cells observed in reactor with medium sparged with CO_2 _instead of N_2 _are in agreement with others studies reporting the essential role of CO_2_, even at low level, to stimulate growth of bifidobacteria [30,32]. Presence of residual dissolved CO_2 _in MRS medium for liquid shaking culture, can also explain the lack of difference between growth of HPR2 and wild type cells in presence of 100% N_2 _or CO_2 _in the headspace, in contrast to cultures in reactor where these gases were directly sparged into the medium.

A known mechanism influencing sensitivity of Bifidobacteria to oxygen is the type of NADH oxidase activity. In O_2 _sensitive species, NADH oxidase exhibits H_2_O_2 _forming activity while H_2_O is produced in microaerophilic species [6,31]. *B. longum *NCC2705 possess a gene (*BL1266*), which codes for a putative NADH oxidase with an active site for the four electron reduction of O_2 _to H_2_O and could therefore be implied in the detoxification of H_2_O_2 _[14]. However, *BL1266 *is not differentially expressed in HPR2 compared to wild type cells. Constitutive overproduction of oxidative stress related proteins can also protect cells to H_2_O_2 _as observed in a mutant strain of *Bacteroides fragilis *resistant to H_2_O_2 _[33]. In HPR2 another mechanism seems to be involved in the resistance to H_2_O_2_. Two genes, *BL1404 *and *BL0931*, were constitutively overexpressed in HPR2 compared to wild type cells. These two genes were not differentially expressed in wild type cells exposed to H_2_O_2 _[14]. BLAST search [34] showed that BL1404, which is annotated as hypothetical protein [35], is highly homologous, 99% on nucleotide level, to the integral membrane protein BLD_0271 from *B. longum *DJ010A. The BLAST search could not identify homologous genes in other bacteria, indicating that *BL1404 *encodes a protein specific to bifidobacteria. The BL1404 protein possesses three predicted transmembrane domains and 2 outer and 2 inner domains. BL0931 is annotated as possible ABC-type transport system involved in lipoprotein release. The functional predictions of the two differentially expressed genes do not suggest any mechanistic explanation for the resistance phenotype of the HPR2 isolate. For this purpose further experiments are required. Transcriptional profiling in the presence of H_2_O_2 _might be more informative for characterization of the resistance phenotype. Moreover, transcriptional profiling analysis is known to be useless for mutations that do not impact gene expression. Therefore, for future experiments, whole genome sequencing using next-generation sequencing technologies will most likely provide better mechanistic insight.

## Conclusions

Our study showed that continuous culture with cell immobilization is a valid approach for selecting cells adapted to hydrogen peroxide. Cell immobilization allowed maintaining high cell numbers in the reactor, even when high selective pressure was applied. This enabled controlled application of stress at high levels on the culture over a long time. Additionally, preliminary characterization of HPR2 revealed the constitutive induction of two genes associated with the cell membrane. However, their function needs further characterization. Elucidation of H_2_O_2 _resistance mechanisms in HPR2 could be helpful to improve resistance of bifidobacteria to oxidative stress.

## Competing interests

Nestec S.A partly supported this project and applied for a patent for the hydrogen peroxide resistant strain.

## Authors' contributions

VM carried out the experimental part and contributed to draft the manuscript. FG participated to the design of the experiments and drafted the manuscript. DM performed all microarray experiments. BB and ER provided essential inputs for the microarray analysis and together with CL, FA and FG, conceived the initial approaches. LM participated to the analysis of the genomic data and to draft the manuscript. CL acted as overall supervisor and corresponding author of the work. All authors have read and approved the final version of the manuscript.
